# YOLOv8 Algorithm-aided Detection of Rib Fracture on Multiplane Reconstruction Images

**DOI:** 10.2174/0115734056337623250212052347

**Published:** 2025-03-17

**Authors:** Shihong Liu, Wei Zhang, Gang Wu

**Affiliations:** 1 Department of Radiology, Fuyong People's Hospital, Shenzhen, Guangdong, China; 2 Department of Radiology, Huazhong University of Science and Technology Tongji Medical College Tongji Hospital, Wuhan, Hubei, China

**Keywords:** Artificial intelligence, Computer vision, Deep learning, Multiplane reconstruction, Rib fracture, YOLOv8

## Abstract

**Objective:**

This study aimed to develop and assess the performance of a YOLOv8 algorithm-aided detection model for identifying rib fractures on multiplane reconstruction (MPR) images, addressing the limitations of current AI models and the labor-intensive nature of manual diagnosis.

**Methods:**

Ethical approval was obtained, and a dataset comprising 624 MPR images, confirmed by CT, was collected from three regions of Tongji Hospital between May 2020 and May 2023. The images were categorized into training, validation, and external test sets. A musculoskeletal radiologist labeled the images, and a YOLOV8n model was trained and validated using these datasets. The performance metrics, including sensitivity, specificity, accuracy, precision, recall, and F1 score, were calculated.

**Results:**

The refined YOLO model demonstrated high diagnostic accuracy, with sensitivity, specificity, and accuracy rates of 96%, 97%, and 97%, respectively. The AI model significantly outperformed the radiologist in terms of diagnostic speed, with an average interpretation time of 2.02 seconds for 144 images compared to 288 seconds required by the radiologist.

**Conclusion:**

The YOLOv8 algorithm shows promise in expediting the diagnosis of rib fractures on MPR images with high accuracy, potentially improving clinical efficiency and reducing the workload for radiologists. Future work will focus on enhancing the model with more feature learning capabilities and integrating it into the PACS system for human-computer interaction.

## 1 INTRODUCTION

Among all fractures, rib fractures are the most difficult to diagnose [1] due to the large number of ribs and the pronounced curvature of their path. Rib CT is currently the best tool for diagnosing rib fractures, far superior to X-ray [2]. However, there are hundreds of thin layer images generated by rib CT, and there were several or more rib segments on each transverse image. Therefore, it is very time-consuming and laborious for doctors to detect rib fractures using the original images of rib CT [2, 3]. Currently, there are some AI models available to assist in the diagnosis of rib fractures [4-7], which were trained based on the original rib images. Multiplane reconstruction (MPR) images of ribs are often indispensable for accurate diagnosis of rib fractures. However, there is currently a lack of AI models trained on rib MPR images, and the current models do not perform well in the detection of rib fractures when using MPR images only. In recent years, computer vision has been used to assist in medical imaging diagnosis with encouraging results [8-11]. The You Only Look Once version 8 (YOLOv8) neural network is an example of deep learning (DL) algorithms which have shown excellent performance in disease detection and diagnosis [12]. The purpose of this study is to refine a YOLOv8 model based on MPR images and determine the accuracy in the detection of rib fractures.

With the rapid development of artificial intelligence technology, the fields of deep learning and computer vision have achieved remarkable results that have attracted worldwide attention. Convolutional neural networks (CNN) and YOLO series models play a crucial role in the critical task of object detection.

A convolutional neural network is a special neural network structure designed specifically for processing image data. It achieves efficient extraction of image features by simulating the hierarchical information processing mechanism of the human visual cortex. Convolutional neural networks are mainly composed of convolutional layers, pooling layers, and fully connected layers. The convolutional layer performs convolution operations on the input image through a series of learnable convolution kernels to extract local features from the image. The pooling layer reduces the dimensionality and complexity of data through down-sampling operations. Finally, the fully connected layer maps the features onto the final classification or regression task.

YOLO (You Only Look Once) is a groundbreaking real-time object detection algorithm. It abandons the complex process in traditional object detection algorithms and transforms the object detection problem into a regression problem, which can predict the position and category of the target through a forward propagation process. This concise and efficient design enables YOLO to achieve a good balance between speed and accuracy. With the continuous advancement of technology, the YOLO series models are also constantly growing and expanding. From the initial YOLOv1 to the current YOLOv8, each generation of models has been optimized and improved on the basis of previous generations. Among them, YOLOv8, as the latest member, further enhances the accuracy of detection while maintaining real-time performance. It adopts a new backbone network, detection head, and loss function, making the model more adept at dealing with various complex scenarios. On the basis of inheriting the real-time detection characteristics of the YOLO series, the YOLOv8 model has comprehensively optimized and improved its structure. It adopts a more efficient backbone network and improves feature extraction capability by introducing new convolutional layers and connection methods. Meanwhile, YOLOv8 also adopts a new detection head design, making the model more accurate and reliable in predicting target positions and categories. In addition, YOLOv8 has further improved the performance of the model by improving the loss function and optimizing the training strategy. In practical applications, the YOLOv8 model has demonstrated excellent real-time performance and accuracy. Whether in autonomous driving, video surveillance, or other scenarios that require real-time object detection, YOLOv8 can quickly and accurately identify target objects, providing strong support for practical applications.

## 2 METHODS AND MATERIALS

The study has been approved by the Institutional Review Board of the university, and informed consent was obtained from patients before the study. The inclusion criteria were patients with rib fractures confirmed by CT. During the model developing stage, the MPR images without rib fractures were excluded from the study. In consideration of the distribution balance among the 24 ribs, 20 MPR images were selected for each rib.

### 2.1 Data Collection

We collected 624 rib MPR images with fractures from Tongji Hospital, covering three locations: the Main Hospital, Sino-French New City Hospital, and Optics Valley Hospital (Centers A, B, and C, respectively), between May 2020 and May 2023. This included 240 images from Center A, 240 from Center B, and 144 from Center C. The images from centers A and B were used for model development, while the images from center C were used for external tests. The images were copied from the medical system, and were saved in JPG format. The 144 images from center C included 72 images with rib fractures and 72 images without rib fractures. There were three images for each rib, so there were 72 images for 24 ribs. The 480 images from centers A and B all had rib fractures.

Two musculoskeletal radiologists with 5 years of clinical experience labeled the 480 images from centers A and B using the software Labelme (https://github.com/CSAILVision/Label MeAnnotationTool). They were asked to draw rectangles to cover all the fractures on the rib, including total fracture and incomplete fracture. The label file ended with “JSON”, and was then converted to a txt file for the purpose of model training.

### 2.2 Model Training

The YOLOV8 model (6.096M size) was used in the study for the 480 labeled images from centers A and B, with a standardized input image size of 640×640. The images were randomly divided into a training set and a validation set according to 8:2. One hundred twenty epochs of training were performed in the study on an NVIDIA GeForce RTX 3060 with 12M memory.

The refined YOLO model obtained after training was further validated by using the 144 images from center C. The artificial intelligence (AI) diagnosis for the images was compared to the ground truth. The numbers of true positives (TP), false positives (FP), true negatives (TN), and false negatives (FN) and time for diagnosis were recorded. Two radiologists with 5 years of experience were asked to diagnose the 144 images from center C, and the time was recorded.

Specificity=TN/(TN+2FP)

Recall (Sensitivity) = TP/ (TP +FN)

Precision= TP/ (TP + FP)

Accuracy= (TP + TN)/ (TP + FP + TN + FN)

F1 score = (2 × Precision × Recall)/ (Precision + Recall)

### 2.3 Statistical Analysis

All statistical calculations were performed using SPSS 26.0 software (SPSS Inc. Chicago, Illinois, USA). The paired t-test was used to analyze the two groups of paired continuous variables. A p-value of less than 0.5 was considered statistically significant.

## 3 RESULTS

**Fig. (1):**
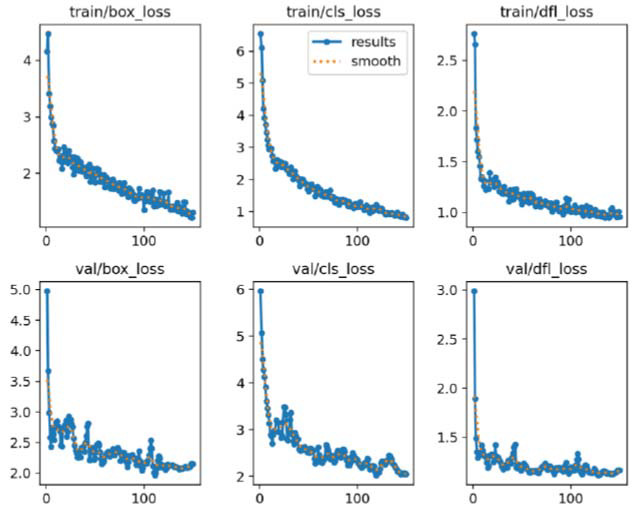
The training process of YOLOv8 model. The graphs depict the decrease of box loss, classification loss and Distribution Focal Loss with epochs. The upper lines and lower lines are for training cohort and validation cohort respectively.

**Fig. (2):**
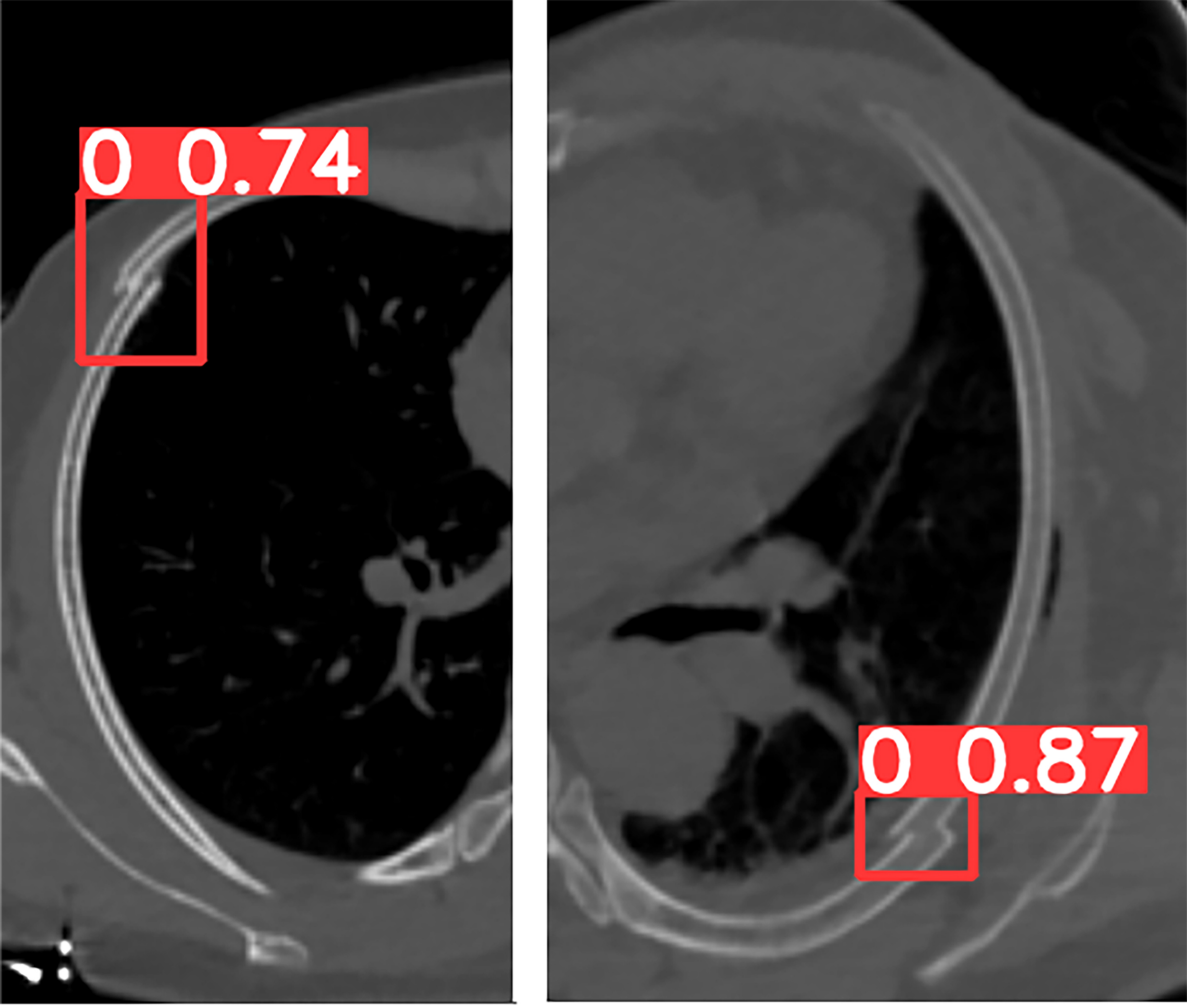
Example of using the refined YOLOv8 model to predict images. 0 represents fracture.

A total of 624 MPR images from 247 participants were included in this study. The 624 images were divided into a training set (n = 384), a validation set (n = 96), and an external test set (n = 144). In the external datasets (including 72 positive), the sensitivity/specificity/accuracy of the refined YOLO model were (96%, 97%, 97%) respectively. The AI model took about 14.01±10.34 ms to interpret each image, and the total time for 144 images was about 2.02 seconds. The radiologist took about 1.3~2.5 seconds to interpret each image, and the total time was 288 seconds. The diagnosing time was significantly shorter for the AI model (P<0.001). In addition, the refined YOLO model has a recall rate of 96%, a precision of 97%, and an F1 score of 0.965. With the increase of training epochs, the reduction of box loss, classification loss, and distribution focal loss (Fig. **1**). We demonstrated an example of predicting images using the refined YOLOv8 model (Fig. **2**).

## 4 DISCUSSION

This study explores the feasibility of using computer vision to identify rib fractures on MPR images. The main findings were: 1) the accuracy of the refined YOLO model in detecting rib fracture was 97%; 2) the diagnostic speed of AI was superior to humans.

Rib fracture is the most common thoracic trauma [8]. Any impact on the chest, including accidents, falls, collisions, *etc*., can potentially cause rib fractures. In practical medical scenarios, there are numerous patients with rib fractures, resulting in a vast amount of imaging data. A patient produces hundreds of chest CT thin-layer images per examination. Typically, a doctor processes a large number of chest CT images daily, resulting in a heavy workload. In addition, many rib fractures occur at night, and doctors need to determine the location of the fracture in the rib as soon as possible. Complex scenarios, such as traffic accidents and judicial appraisals, require flawless fracture detection, placing significant demands on doctors. AI is therefore crucial in the clinical practice of rib fracture detection [9].

In the study, we developed a rib-fracture AI model based on the datasets of MPR images but not the original transverse images. It has been well established that MPR images are more valuable than original images for the detection of rib fractures. However, MPR images could not be generated by the CT scanner automatically but were created by the experienced radiologists on the dedicated workstation. Generally, there were 24 or more MPR images for each patient, and only a few of them had rib fractures. Thus, it is not easy to collect hundreds of MPR images with rib fractures. Fortunately, in the author’s center, MPR images are routinely generated for the examination of rib CT.

Our rib image datasets are balanced among 24 ribs, thus, the model obtained in the study can accurately detect fractures of all ribs. Rib fractures were divided as incomplete fracture, complete fracture and old fracture in the study. They were labeled as different types when using the Labelme tool. Another method is to label all these fractures as only one type (fracture). However, this method will produce a model incapable of classifying fracture types.

Although YOLOv8 was proposed by Ultralytics in 2023, we used the algorithm for the first time to examine it. YOLOv8 builds on the previous versions of the YOLO series with a new backbone network, a new Ancher-Free detection head, and a new loss function, making the model perform well in terms of speed and accuracy. The architecture of YOLOv8 consists of a backbone network designed to extract features from the input image and a head network that predicts bounding boxes and object classification [10, 11].

YOLO model is a friendly-to-user AI tool that appears to reduce diagnosis time and improve accuracy. Radiologists can further analyze the images previously processed by computer vision and ultimately improve the diagnostic efficiency. Our future work will focus on developing a human-computer interaction platform based on the YOLOv8 model and integrating it into the PACS system.This study has some limitations. First, the sample size is small, and larger studies are needed to validate our findings. Second, only a simple dichotomous model construction was carried out, and a multi-categorical model was not constructed because the sample size of abnormal images was too small. Third, only the YOLOv8 model was used, and other computer visual models, such as two-stage RCNN, were not used, and the diagnostic performance of each model could be compared in the future.

## CONCLUSION

In this multi-center study, the refined YOLOV8n model demonstrated significantly enhanced performance in real-time object detection compared to its predecessors. In the future, the model will be further improved by incorporating more feature-learning capabilities to achieve better inspection performance.

## AUTHORS’ CONTRIBUTION

Shihong Liu, Wei Zhang, and Gang Wu researched literature and conceived the study. Shihong Liu wrote the first draft of the manuscript. All authors reviewed and edited the manuscript and approved the final version of the manuscript.

## Data Availability

The data and supportive information are available within the article.

## LIST OF ABBREVIATIONS

ROI: Region of interest
YOLOv8: You Only Look Once version 8
DL: Deep Learning
AI: Artificial Intelligence
